# Bioelectrochemistry for flexible control of biological processes

**DOI:** 10.1016/j.ese.2020.100011

**Published:** 2020-01-11

**Authors:** Annemiek ter Heijne

**Affiliations:** Environmental Technology, Wageningen University & Research, Bornse Weilanden 9, 6708 WG, Wageningen, the Netherlands

Societal awareness and worries about the future of the earth are rapidly increasing. Who does not want to contribute to a sustainable future, in which the earth’s resources will still be available for future generations?

In my view, a sustainable world is a world in which we live in close collaboration with nature. In nature, microorganisms are a key player in the global carbon, nitrogen and phosphorous cycle. Therefore, these natural processes are the key to sustainable solutions for environmental problems, like the recovery of resources from waste streams, sustainable energy generation, and degradation of pollution. The main challenge of these biological conversions is to engineer them towards the desired rate and efficiency, to obtain a sustainable and effective technology.

Currently, biotechnological processes are widely applied in wastewater and gas treatment for removal and recovery of organic material, nitrogen, phosphate and sulphur. All these biological conversions consist of combinations of reduction and oxidation reactions. Measurement of Oxidation-Reduction Potential (ORP) that reflects the availability of electron donors and acceptors in solution is used as an operational parameter for steering selectivity and stability of these biological processes. Each process has its own ORP range, in which the conversion is most effective (Table 1) [1].

**Table 1:** Overview of the ORP range in which different biological processes typically occur.

Biochemical Activity	ORP Range (mV)
Aerobic carbon oxidation	+50 to +200
Nitrification	+150 to +350
Denitrification	−50 to +50
Acidification	−200 to −40
Sulphate reduction	−250 to −50
Methanogenesis	−400 to −200

Supply of electron donor and acceptor is the common strategy to control the redox potential. For example, to remove organic carbon from wastewater, the supply of air is controlled to keep the desired ORP. Based on ORP measurements alone, however, it is challenging to precisely control the biological conversions, particularly when specific performance is needed. The reason is that ORP is a lumped parameter that reflects the presence of all oxidizing and reducing components in the environment. As a result, ORP based control does not always result in high selectivity of biological conversions. In addition, the potential range in which redox conditions can be tuned with ORP is limited.

To overcome the challenge that electron donor and/or acceptor are often limiting biological conversions, electrodes can be used as an additional source or sink for electrons. Electrodes can be used to increase the operating range of biological conversions because the electrode potential can be precisely controlled at any oxidizing or reducing condition. As many different bacteria can exchange electrons with an electrode [[2], [3], [4]] - so-called electroactive bacteria - electrodes provide a unique platform for the study and control of microbial conversions. These electroactive bacteria commonly live in biofilms that are attached to the electrode. This interaction between electrodes and bacteria forms the basis of Microbial Electrochemical Technologies (METs) [5,6].

In the early 2000s, the first principles of Microbial Fuel Cells (MFCs) were demonstrated [7]. In the following decade, METs have gained much attention as a new, sustainable technology to convert chemical energy into electrical energy or vice versa. Potential applications are the recovery of energy and nutrients from wastewater, and the conversion of electricity into fuels and chemicals [6]. It was, for example, discovered that bacteria could take up electrons (or reducing equivalents in the form of hydrogen) from a cathode [8], thereby reducing CO_2_ to products like methane [9,10], acetate [11], and medium-chain fatty acids [12,13]. This discovery opened new opportunities for the conversion of electricity and CO_2_ into added-value components using microorganisms.

In the coming decades, many new applications will arise that are based on the exchange of electrons between microorganisms and electrodes, both from a control and a sustainability perspective. One important reason that electrodes will play a more dominant role in our society in the future is the rapidly developing energy transition. As we will become more and more reliant on renewable electricity in our energy mix, processes that can exchange electrical energy for chemical energy in an efficient way will become more important. A setback of many electrochemical processes today is that they are not truly sustainable, since they require scarce and expensive catalysts, and they do not occur at ambient conditions. This is where electroactive microorganisms come in – they are the potentially more sustainable alternative.

So what will biological processes and electrochemistry in 2050 look like? And what is needed to get there?

In 2050, many biological processes, for example, for organic matter and nutrient removal, will not rely anymore on aeration. Electrodes will have taken over the role of oxygen as an electron acceptor. Biological desulphurization is one of those processes where aeration could be replaced by electrodes: we have recently shown that aerobic sulphide oxidizing bacteria can remove sulphide from solution under anaerobic conditions, and can shuttle the electrons to an electrode in an electrochemical cell [14]. In 2050, many of these installations will thus use electrodes rather than oxygen, not only to produce elemental sulphur from sulphide but also to recover energy from the process, rather than requiring energy for aeration. Many other ‘conventional’ biological processes that use aeration could thus be replaced by (bio)electrochemical processes, with the advantage of creating more energy-efficient conversion processes. In addition to replacing the electron acceptor oxygen with an electrode, other biological processes will be developed that use electrodes as flexible electron donor control. Use of METs as a sensor to monitor and control biological conversions is another promising application [15].

One of the key challenges for the use of electrodes to influence biological conversions is electron efficiency. It is essential that a large part of the electrons from the electron donor end up in the desired product (high selectivity), to make electrodes a sustainable alternative electron donor or acceptor. These electron efficiencies, from an electron donor to acceptor, are currently highly variable. For bioanodes, electron efficiency is usually much higher for acetate as electron donor compared to complex organic substrates [16]. For biocathodes, especially when methane is the final product, electron efficiencies range from 20 to 100% [17]. When electron balances do not add up to 100%, part of the electrons is lost to unwanted products hydrogen, oxygen, methane, or microbially produced intermediates like formate, that decrease the overall efficiency of the conversion. In addition to electron efficiency, also the voltage efficiency is a key performance parameter for METs [18]. The voltage efficiency is influenced by the overpotentials that occur at the anode and the cathode. With regard to this overpotential, the interaction between electrode potential and biological activity is currently not well-understood, and there is little information on the growth kinetics of electroactive biofilms as a function of these electrode potentials. Detailed insight into the effect of electrode potential on electroactive biofilm activity is crucial for the integration of electrodes in biological conversions. To be able to use electrodes as a flexible control strategy for biological processes.

In many biological conversions, hydrogen is an intermediate component or a by-product. Whereas some biological conversions, like acidification, require very low hydrogen partial pressures, other biological conversions, like hydrogenotrophic methanogenesis, rely on hydrogen as the energy source [19]. Interspecies hydrogen transport and direct interspecies electron transport are key processes that influence the rate and selectivity of biological processes. Electrodes can be used to scavenge hydrogen (or electrons) from microorganisms at the anode, to provide additional hydrogen (or electrons) to microorganisms at the cathode, but also as conductive material to promote direct interspecies electron transport, without an external electric circuit [20].

In conclusion, electrodes offer many exciting opportunities for a new, flexible way to control biological conversions. Many scientific and engineering challenges remain. The high versatility of reactions and applications is the key to success of METs: niches need to be identified where METs will indeed be a more sustainable and economical alternative to conventional (bio)processes. I am excited to further contribute in this field of METs with research, scaling-up and pilot testing, to contribute to a more sustainable world.

## References

[1] Sleutels T., Molenaar S., Heijne A., Buisman C. (2016). Low substrate loading limits methanogenesis and leads to high Coulombic efficiency in bioelectrochemical systems. Microorganisms.

[2] Kracke F., Vassilev I., Krömer J.O. (2015). Microbial electron transport and energy conservation - the foundation for optimizing bioelectrochemical systems. Front. Microbiol..

[3] Reguera G., McCarthy K.D., Mehta T., Nicoll J.S., Tuominen M.T., Lovley D.R. (2005). Extracellular electron transfer via microbial nanowires. Nature.

[4] Lovley D.R. (2012). Electromicrobiology. Annu. Rev. Microbiol..

[5] Schröder U., Harnisch F., Angenent L.T. (2015). Microbial electrochemistry and technology: terminology and classification. Energy Environ. Sci..

[6] Hamelers H.V.M., Ter Heijne A., Sleutels T.H.J.A., Jeremiasse A.W., Strik D.P.B.T.B., Buisman C.J.N. (2010). New applications and performance of bioelectrochemical systems. Appl. Microbiol. Biotechnol..

[7] Bond D.R., Lovley D.R. (2003). Electricity production by geobacter sulfurreducens attached to electrodes. Appl. Environ. Microbiol..

[8] Nevin K.P., Woodard T.L., Franks A.E., Summers Z.M., Lovley D.R. (2010). Microbial electrosynthesis: feeding microbes electricity to convert carbon dioxide and water to multicarbon extracellular organic compounds. MBio.

[9] Cheng S., Xing D., Call D.F., Logan B.E. (2009). Direct biological conversion of electrical current into methane by electromethanogenesis. Environ. Sci. Technol..

[10] Van Eerten-jansen M.C.A.A., Heijne A. Ter, Buisman C.J.N., Hamelers H.V.M. (2012).

[11] Jourdin L., Freguia S., Donose B.C., Chen J., Wallace G.G., Keller J., Flexer V. (2014). A novel carbon nanotube modified scaffold as an efficient biocathode material for improved microbial electrosynthesis. J. Mater. Chem. A.

[12] Van Eerten-Jansen M.C.a. a., Ter Heijne A., Grootscholten T.I.M., Steinbusch K.J.J., Sleutels T.H. J.a., Hamelers H.V.M., Buisman C.J.N. (2013). Bioelectrochemical production of caproate and caprylate from acetate by mixed cultures. ACS Sustain. Chem. Eng..

[13] Angenent L.T., Richter H., Buckel W., Spirito C.M., Steinbusch K.J.J., Plugge C.M., Strik D.P.B.T.B., Grootscholten T.I.M., Buisman C.J.N., Hamelers H.V.M. (2016). Chain elongation with reactor microbiomes: open-culture biotechnology to produce biochemicals. Environ. Sci. Technol..

[14] ter Heijne A., de Rink R., Liu D., Klok J.B.M., Buisman C.J.N. (2018). Bacteria as an electron shuttle for sulfide oxidation. Environ. Sci. Technol. Lett..

[15] Kim B.H., Chang I.S., Gil G.C., Park H.S., Kim H.J. (2003). Novel BOD (biological oxygen demand) sensor using mediator-less microbial fuel cell. Biotechnol. Lett..

[16] Hamelers H.V.M., Ter Heijne A., Sleutels T.H.J.A., Jeremiasse A.W., Strik D.P.B.T.B., Buisman C.J.N. (2010). New applications and performance of bioelectrochemical systems. Appl. Microbiol. Biotechnol..

[17] Geppert F., Liu D., van Eerten-Jansen M., Weidner E., Buisman C., Ter Heijne A. (2016). Bioelectrochemical power-to-gas: state of the art and future perspectives. Trends Biotechnol..

[18] Sleutels T.H.J.A., TerHeijne A., Buisman C.J.N., Hamelers H.V.M. (2012). Bioelectrochemical systems: an outlook for practical applications. ChemSusChem.

[19] Stams A.J.M., de Bok F.A.M., Plugge C.M., van Eekert M.H.A., Dolfing J., Schraa G. (2006). Exocellular electron transfer in anaerobic microbial communities. Environ. Microbiol..

[20] Martins G., Salvador A.F., Pereira L., Alves M.M. (2018). Methane production and conductive materials: a critical review. Environ. Sci. Technol..

